# Managing Cardiac Resynchronization Therapy Nonresponse: Conventional and Unconventional Techniques

**DOI:** 10.19102/icrm.2018.091107

**Published:** 2018-11-15

**Authors:** Richard G. Trohman, Henry D. Huang, Ryan M. Zimberg, Nicholas J. Serafini, Parikshit S. Sharma

**Affiliations:** ^1^Division of Cardiology, Department of Internal Medicine, Rush University Medical Center, Chicago, IL, USA

**Keywords:** Cardiac resynchronization therapy, catheter ablation to enhance CRT, multipoint pacing, multisite pacing, permanent His-bundle pacing

## Abstract

Cardiac resynchronization therapy (CRT) is an established option for select patients with systolic heart failure. Nonresponse to CRT remains a significant problem. We present five patients who were CRT nonresponders and discuss the conventional and nonconventional approaches employed to achieve CRT benefit.

## Introduction

Cardiac resynchronization therapy (CRT) is applicable in 25% to 30% of patients with symptomatic heart failure (HF). Patients with left bundle branch (LBB) block (LBBB) and QRS duration ≥ 150 ms seem to derive the greatest benefit from CRT. Current scientific evidence suggests that approximately 30% of the patients who are selected for CRT do not respond to this therapy.^1^ However, there is a lack of standard definition for CRT response and the spectrum of CRT response and reported response rates vary widely depending on the metrics used and whether or not a placebo effect is considered.^2–4^ Regardless of the definition considered, however, nonresponse to CRT remains a significant problem. This manuscript presents five cases of nonresponse to CRT and will discuss the approaches used to overcome this problem in each.

## Case presentations

### Case 1

A 72-year-old male with HF and ischemic cardiomyopathy with a left ventricular (LV) ejection fraction (LVEF) of 25% and LBBB was referred for CRT defibrillator (CRT-D) implantation. Three months following implantation, he felt dramatically better. His percentage of biventricular (BiV) pacing was 95% and his LVEF had improved to between 45% and 50%.

Nine months later, he returned for a follow-up visit and reported feeling more symptomatic with regard to HF. Further evaluation revealed that his LVEF had dropped to between 25% and 30%. The percentage of BiV pacing was noted to be 78% and a 12-lead electrocardiogram (ECG) revealed uniform monomorphic premature ventricular complexes (PVCs) in a bigeminal pattern. A three-day ambulatory monitor revealed a 38% PVC burden. He was uninterested in entertaining antiarrhythmic drug options and decided to undergo an attempt at PVC ablation.

Electrophysiologic testing and mapping of his PVCs revealed a focus at the mid-right ventricular (RV) septum. Ablation at this site eliminated his PVCs but was complicated by complete atrioventricular block. When he returned for additional follow-up three months later, he reported marked symptomatic improvement. His LVEF had returned to between 45% and 50% and his percentage of BiV pacing was 99%.

### Case 2

**Figure 1:**
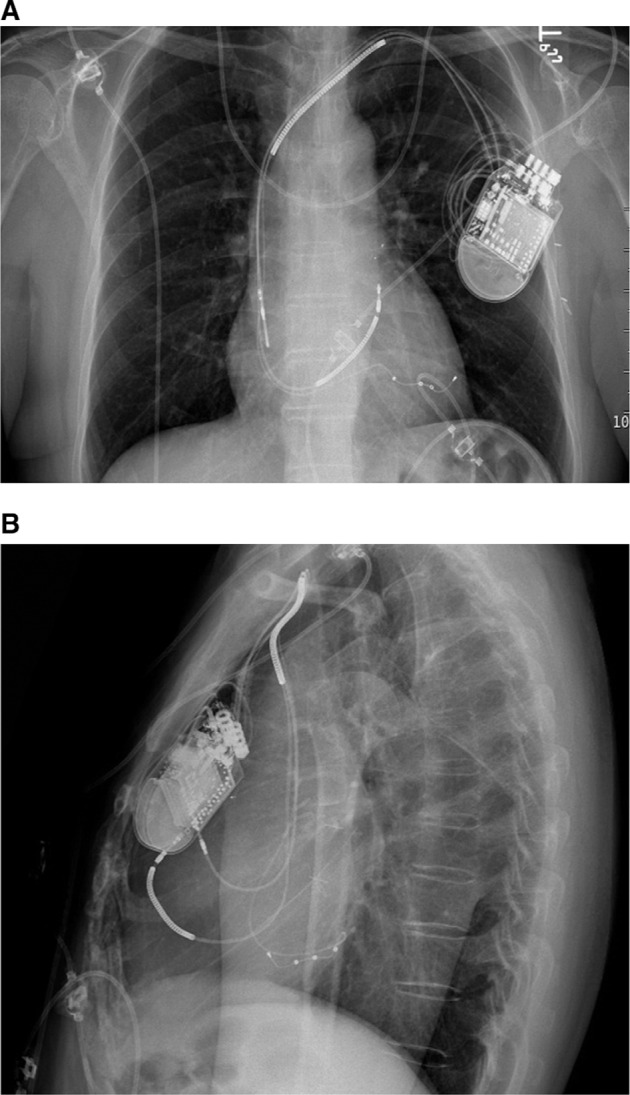
Posteroanterior **(A)** and lateral **(B)** chest X-rays demonstrating the ICD lead in the anteroseptal position that resulted in para-Hisian pacing.

**Figure 2:**
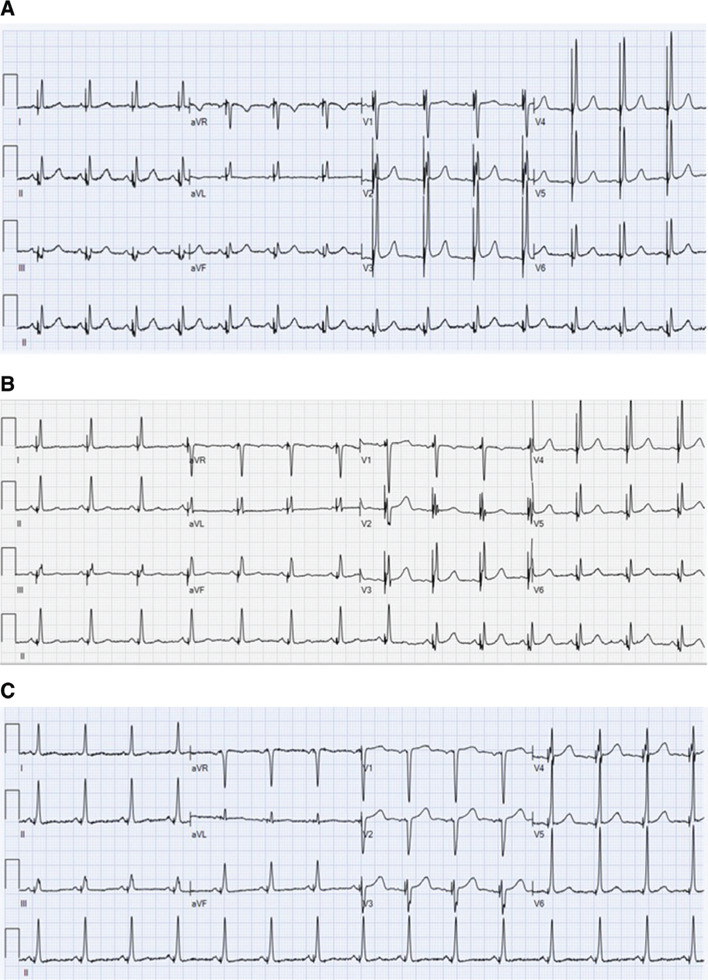
ECGs following RV lead repositioning. **A:** BiV pacing is present with a narrow QRS complex. **B:** After the LV pacing threshold rose, intermittent LV capture was present only during the final six beats and is best seen in the lead II rhythm strip. **C:** After LV pacing is turned off, para-Hisian pacing remains as a narrow QRS complex.

A 64-year-old female with a history of nonischemic cardiomyopathy, LBBB, and an LVEF of 10% to 15% underwent CRT-D implantation. Her QRS duration decreased from 136 ms to 126 ms. She reported being bothered by diaphragmatic stimulation and, despite 99% BiV pacing, an echocardiogram performed at five months after the procedure still revealed an LVEF of 10% to 15%. Her apically placed RV lead capture threshold increased to 5.25 V per 1.5 ms and a decision was made to revise the RV lead. The lead in the RV apex was removed with gentle manual traction. A new RV defibrillation lead was placed in the anteroseptal region **(Figure 1)** and defibrillation threshold testing revealed a safety margin of more than 10 J. Following lead revision, her QRS duration decreased to 104 ms **(Figure 2A)**.

When the patient returned for follow-up at six months after her previous echocardiogram, she reported symptomatic improvement and her LVEF had increased to between 30% and 35%. During subsequent follow-up, her ejection fraction rose slightly to 35% to 40%.

At this point, her LV pacing threshold increased and adjustments to higher outputs resulted in uncertain capture and more diaphragmatic stimulation. A 12-lead ECG confirmed intermittent LV capture **(Figure 2B)**. Nevertheless, in the absence of LV capture, the QRS remained remarkably narrow. When LV pacing was turned off, her QRS width remained at 104 ms and her diaphragmatic stimulation disappeared **(Figure 2C)**. To date, the patient has continued to do well in the absence of LV pacing. It seems certain that her clinical improvement resulted from resynchronization via para-Hisian pacing and the recruitment of fibers distal to the site of original disease in the LBB, and that this outperformed conventional CRT.

### Case 3

A 55-year-old male with a nonischemic cardiomyopathy with an LVEF of less than 20% underwent CRT-D placement. He had a history of atrial fibrillation (AF) with rapid rates resistant to digoxin and several different ß-blockers. He was transferred from an outside hospital to undergo management of poorly controlled AF and HF. His LVEF remained low and his percentage of BiV pacing was only 2.7%. Given his AF with rapid ventricular response refractory to medical therapy and ineffective BiV pacing, the patient underwent an atrioventricular junction ablation.

Following the procedure, his percentage of BiV pacing improved to 99%. He has been asymptomatic to date during follow-up and an echocardiogram performed at 14 months postprocedure revealed an LVEF of 55%.

### Case 4

A 68-year-old man with a history of hypertension, non-obstructive coronary artery disease, and nonischemic cardiomyopathy (LVEF: ~15%) underwent single-chamber implantable cardioverter-defibrillator (ICD) placement in 2007 and successful ablation of typical atrial flutter in 2010. In 2011, he underwent an atrioventricular junction ablation and an upgrade to a dual-chamber ICD for shocks related to AF and rapid ventricular response. Then, in 2015, his LVEF was estimated to be in the range of 25% to 30%. His ICD subsequently reached its elective replacement interval in 2016. At that time, he reported significant functional decline with fatigue and dyspnea on exertion. A decision was made to upgrade him to a CRT-D system.

**Figure 3:**
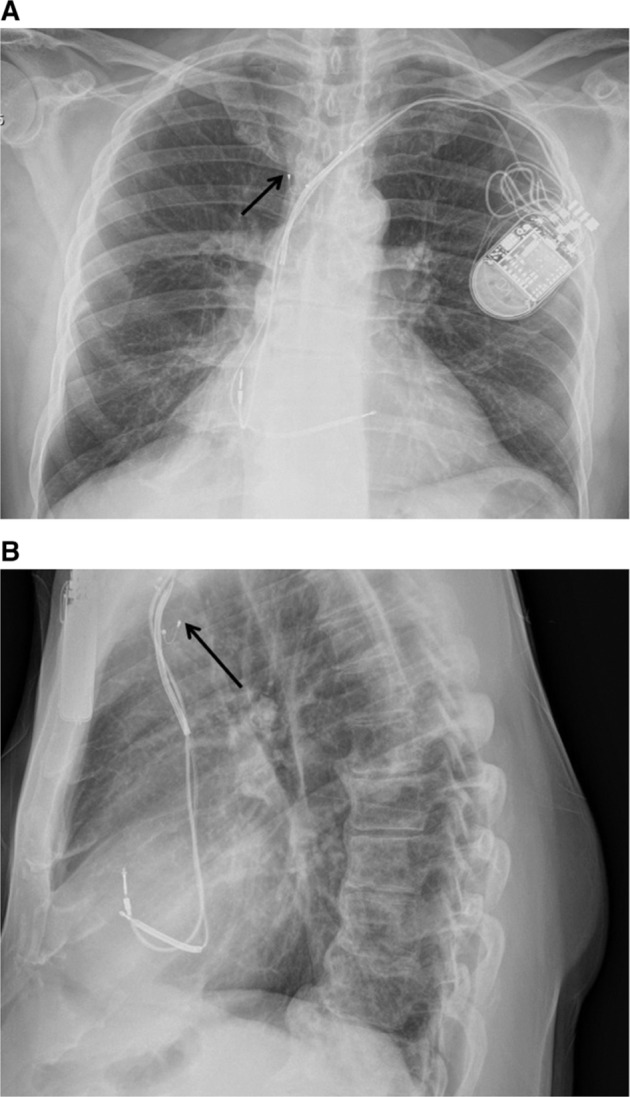
The arrows on the posteroanterior **(A)** and lateral **(B)** chest X-rays demonstrate retraction of the LV lead to the superior vena cava, resulting in phrenic nerve stimulation.

A Quartet™ 1458QL lead (Abbott Laboratories, Chicago, IL, USA) was easily advanced to a large posterolateral cardiac vein. Two weeks later, during a wound/device check, the LV lead failed to capture in any configuration at maximum output. The initial Quartet™ 1458QL lead (Abbott Laboratories, Chicago, IL, USA) was subsequently removed and a new Quartet™ 1458QL lead (Abbott Laboratories, Chicago, IL, USA) was placed in a similar position as the first lead in the posterolateral cardiac vein. Two months later, the patient presented to the clinic while feeling poorly and experiencing diaphragmatic stimulation. The LV lead was noted to have retracted to the superior vena cava **(Figure 3)**.

**Figure 4:**
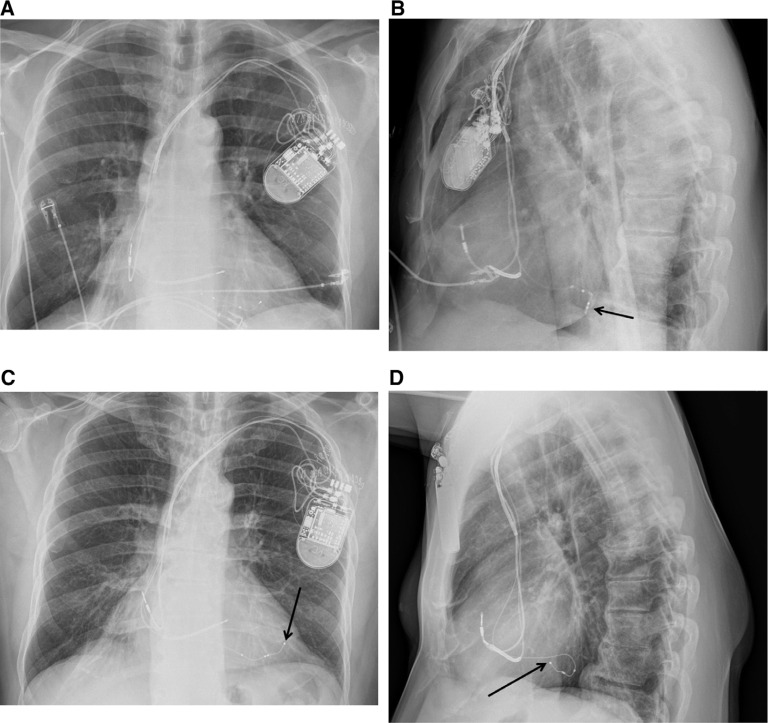
Posteroanterior **(A)** and lateral **(B)** chest X-rays demonstrating the position of the quartet lead. The posteroanterior **(C)** and lateral **(D)** chest X-rays reveal that the canted lead was able to reach a slightly different and more stable position. The arrows indicate the approximate position of the LV lead tip (distal electrode).

The patient returned to the electrophysiology laboratory and the second Quartet™ 1458QL lead (Abbott Laboratories, Chicago, IL, USA) was removed. An Attain^®^ Performa™ 4298 lead (Medtronic, Minneapolis, MN, USA) was subsequently chosen and advanced via the same posterolateral branch further into the inferiorly directed tributary to a slightly more anterior position, which provided long-term lead stability **(Figure 4)**. During one year of follow-up, the patient’s symptoms were markedly better and his LVEF had improved to between 35% and 40%.

### Case 5

A 75-year-old female with a history of transcatheter aortic valve replacement (TAVR), LBBB (QRS duration: 146 ms), and New York Heart Association (NYHA) functional class III HF (LVEF: 45%) underwent CRT pacemaker implantation. During surgery, the RV lead was placed at the midseptum and the LV lead was positioned in a posterolateral cardiac vein. After the operation, the paced QRS duration was 158 ms and, unfortunately, she was deemed a nonresponder. Her LVEF progressively deteriorated over seven months to between 20% and 25% with accompanying symptoms of HF. Given the lack of additional targets for LV lead placement, a lead was added in the His-bundle position without complete LBBB recruitment. A Y-adaptor connected the His and cardiac venous leads to the device’s LV port. Although unipolar His pacing resulted in a QRS duration of 170 ms without left bundle recruitment, simultaneous His–RV pacing resulted in a QRS duration of 130 ms and a loss of LBBB pattern. After six months, her LVEF improvement was modest (30%–35%), but her symptoms had improved to NYHA functional class I.

## Discussion

Although the definitions of CRT response are many and varied, it is clear that the overall survival of nonresponders (50% survival rate at four years after implantation) is comparable to that in patients with various types of malignancies.^5,6^ In a 2014 review, Madias and Trohman lamented that, “although CRT has helped many patients with HF, standard triple-chamber pacing has remained largely unchanged for the past 10 years.”^1^

The causes of nonresponse are multifactorial and include patient selection; anatomic limitations, including coronary sinus branches, the presence of myocardial scar, and phrenic nerve stimulation; inadequate understanding of programming options; lead instability; and a low percentage of BiV pacing due to concomitant arrhythmias. **Table 1** summarizes interventions that may be considered in nonresponders.

In a large cohort from the ALTITUDE clinical science program (Boston Scientific, Natick, MA, USA), mortality was inversely associated with the percentage of BiV pacing, and the longest survival benefit occurred with BiV pacing > 98.5%.^7^ In our case series, the patient in case 1 was originally a CRT responder but developed new ventricular ectopy that reduced his percentage of BiV pacing. Ablation of his PVCs (as well as atrioventricular block) resulted in 99% BiV pacing and restoration of near-normal LV function. This case (as well as case 4) illustrates the importance of treating arrhythmias medically or invasively to achieve CRT benefit.

Despite the availability of quadripolar leads (and multiple pacing configurations), phrenic nerve stimulation remains an impediment to CRT. The patient in case 2 did not respond to conventional CRT-D and suffered from phrenic nerve stimulation. Placement of a defibrillation lead in the anteroseptal region permitted para-Hisian pacing and functional improvement. Our group and others have provided evidence that permanent His-bundle pacing (HBP) is a viable alternative to BiV pacing for CRT and an option when percutaneous CRT is unsuccessful.^8–12^ Although defibrillation was successful in case 2, it remains uncertain whether this technique is safe and reliable for the termination of ventricular tachyarrhythmias in most cases.

The patient in case 2 suffered from rapidly conducting AF, which rendered BiV pacing ineffective. AF occurs in 10% to 25% of patients with NYHA functional class II or class III HF and in up to 50% of patients with functional class IV HF. Atrioventricular junction ablation resulted in highly effective CRT and the restoration of a normal LVEF. It is reasonable to speculate that this patient’s low LVEF could have been tachycardia-mediated and might have been ameliorated by atrioventricular junction ablation alone. Nevertheless, in the presence of established CRT, the optimization of BiV pacing was the clear-cut treatment of choice.

In the Cardiac Resynchronization in AF Patients Multinational Registry (CERTIFY) study, long-term survival rates in patients with permanent AF who underwent atrioventricular junction ablation were similar to those in patients in sinus rhythm. Mortality was higher in patients who received rate-slowing drugs.^1,13^ Physicians reluctant to make patients pacemaker-dependent should be advised that they might be limiting CRT benefits with this decision. The likelihood of sudden simultaneous failure of two ventricular leads is exceedingly low.

The patient in case 4 illustrates the value of repositioning the LV lead and emphasizes that no tool is universally effective in achieving successful CRT. Lead location and stability are pivotal to CRT success and essential in reducing phrenic nerve stimulation and premature battery depletion.

The optimal lead positioning strategy remains controversial.^14^ Reliance on anatomical positioning is appealing to many operators because of its relative simplicity. It remains a common practice to target a lateral or posterolateral cardiac vein.^14^ However, in the Multicenter Automatic Defibrillator Implantation Trial–CRT (MADIT-CRT) study, CRT benefit was similar among leads placed in the anterior, lateral, or posterior positions.^15^ Apical LV lead placement has been associated with poorer CRT outcomes.^14–16^ Achieving maximal anatomical separation (assessed radiographically) between RV and LV leads and/or targeting LV regions with maximal electrical delay may help to improve response rates.^14,17,18^ Targeting LV segments with maximal mechanical dyssynchrony has also been advocated for.^14,19^ Echocardiographic speckle-tracking two-dimensional radial strain imaging has been used to target sites of latest activation and to avoid lead placement in areas of myocardial scar.^1,20^

Pacing scar or regions with very slow conduction is likely to be ineffective (see below). Echocardiography has played additional roles in managing CRT recipients. A variety of Doppler parameters have been used for echo-guided optimization. It has been suggested that better results occur when atrioventricular delay is optimized before changing ventricular–ventricular (V–V) delay. Unfortunately, echo optimization is time-consuming, not carried out consistently in each patient, and performed at rest with the patient reclining. Device algorithms are quicker but not clearly better.^1^

A few studies^21–23^ have demonstrated that “prepacing” or “fusion pacing” (ie, optimized LV pacing in the presence of intact right bundle conduction) may have benefits over BiV pacing in preserving RV function and reducing battery drain. In addition, the potentials for dynamic atrioventricular optimization and for simplifying the optimization process have made this concept attractive. The AdaptivCRT™ algorithm (Medtronic, Minneapolis, MN, USA) assumes that when intrinsic conduction is present, LV prepacing is better and, when intrinsic conduction is delayed (or absent), simultaneous BiV pacing is superior. The algorithm measures the atrioventricular interval each minute (the atrioventricular delay is briefly extended to 300 ms) and automatically updates the atrioventricular delay to optimize CRT (70% of the intrinsic atrioventricular delay and at least 40 ms prior to RV sensing). BiV pacing is initiated when atrial-sensed atrioventricular intervals exceed 200 ms and atrial-paced intervals exceed 250 ms. During BiV pacing, the atrioventricular delay is adjusted to 30 ms after the P-wave and 50 ms before RV-sensed events. V–V optimization delivers LV pacing first if there is fusion and a QRS of ≤ 150 ms; if fusion is absent, simultaneous BiV pacing occurs.^1,21–23^

The patient in case 5 reemphasizes the potential utility of HBP. In the last few years, the availability of better delivery systems has made permanent HBP more feasible. Pacing distal to the site of conduction delay (in patients with LBBB or right bundle branch block) can recruit fibers predestined to be part of the bundle branches and thereby narrow QRS duration. HBP has become an attractive alternative to BiV pacing for CRT.^8,24^ Five small studies have revealed improvements in NYHA functional class and LVEF.^9–12^ A more recent report from a multicenter study also concluded that HBP has emerged as a rescue strategy for failed BiV pacing and may be a reasonable primary alternative to BiV pacing for CRT.^25^

In addition, this case illustrates an unusual approach to multisite pacing to achieve a resynchronization response. Widespread adoption of multisite pacing (by convention using two leads in different cardiac veins or two separate RV sites) should not be advocated for in the absence of convincing, large-scale data. Additionally, this technique is likely to be limited by prolonged procedure times and increased radiation exposure.^26^

**Figure 5:**
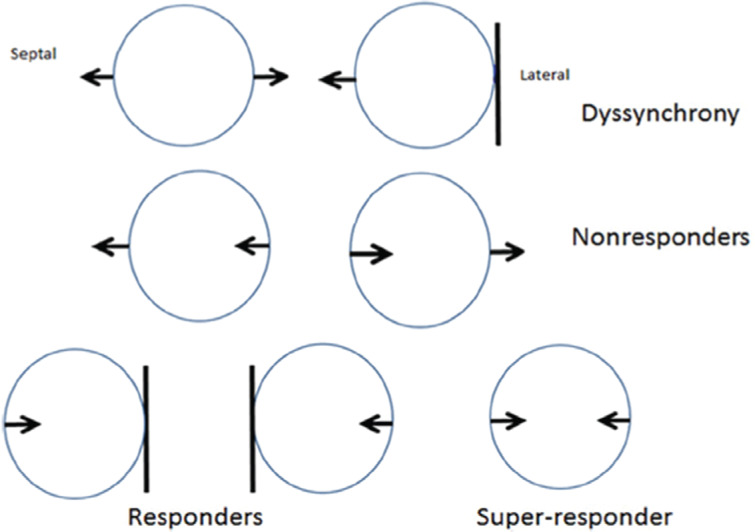
The circles represent the LV chamber. The left side of each circle is septal and the right side is lateral. The vertical septal lines indicate simultaneous activation of the RV and LV septum. The vertical lateral lines indicate akinesis. The arrows indicate the direction of wall motion. The top row demonstrates baseline dyssynchronous LV contraction. The middle row demonstrates activation by LV pacing sequences that would not contribute to enhanced contractility (nonresponse). The bottom row demonstrates two patterns that would improve contractility (response) and one that likely produces synchronized contraction (potential for super-response).

The efficacy of CRT is commonly (and often too simplistically) attributed to a reduction of mechanical inefficiency from dyssynchronous contraction, allowing for more blood to be ejected with lower energy consumption. In responders, CRT specifically reverses many profound basic cellular and molecular changes that occur in dyssynchronous HF.^1^ Nevertheless, simple two-dimensional models **(Figure 5)** provide insight into nonresponders, responders, and super-responders. In these diagrams, a response only occurs when right and left septal activation is simultaneous or when left-sided septal activation is first. If a scenario exists in which early left-sided septal activation is not associated with the elimination of paradoxical lateral wall movement, the likelihood of patient improvement is 60%. If, on the other hand, this scenario is extremely rare or does not occur, then the likelihood of improvement is 75%. A success rate of 60% to 75% fits well with known clinical response rates. We believe that early activation of the left septum is essential to super-response. Data suggesting that LV septal pacing reduces dyssynchrony and preserves LV contractility in comparison with RV septal or apical pacing support the notion that activation of the LV septum prior to the RV septum is likely to contribute to CRT response.^26^

**Figure 6:**
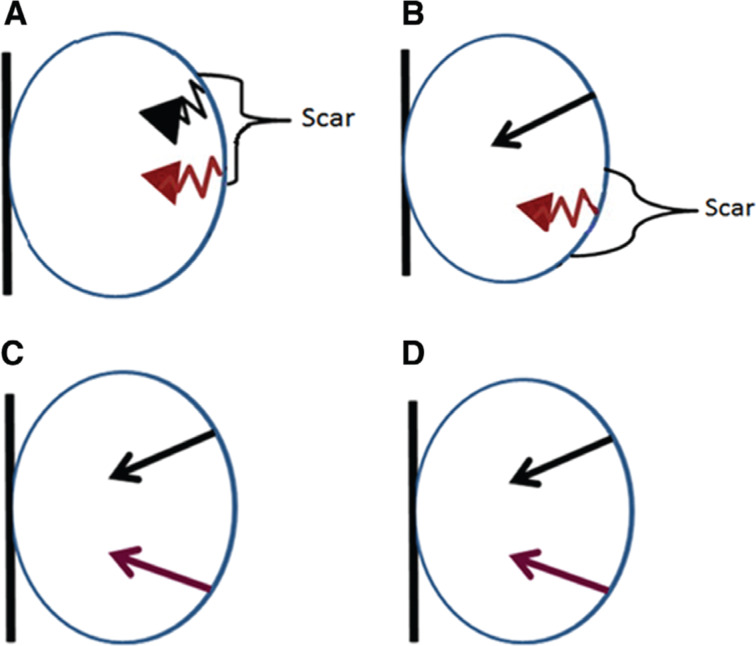
The red arrows demonstrate single-point LV pacing. The black arrows signify the programming of multipoint pacing. The straight arrows indicate depolarization and good conduction. The bent arrows indicate an absence of depolarization, marked slowing, or conduction block. The right and left septum are assumed to contract simultaneously to simplify the scenarios depicted. **A:** Single-point pacing and MPP within scar do not improve contractility. **B:** Single-site pacing within a scar is ineffective, but the use of a second pacing site beyond the scar improves contractility. **C:** Although both pacing sites appear to be effective, their proximity limits the recruitment of additional myocardium. **D:** Increasing the distance between effective MPP sites is likely to recruit more myocardium and improve contractility.

In contrast to multisite pacing, multipoint pacing (MPP) via an LV quadripolar lead is readily achievable. Like multisite pacing, the basic premise behind MPP rests on capturing more volume of the myocardium and increasing the likelihood that areas of (baseline) late depolarization will be captured sooner, thereby resulting in better resynchronization. Ideally, a larger amount of LV is simultaneously depolarized than can be achieved with single-site LV pacing alone. Wide separation (> 30 mm) of the two LV pacing points seems to depolarize more myocardium and reduce the likelihood of both sites pacing scar. Although promising preliminary data are available, the incremental benefit of MPP over conventional CRT remains unclear and awaits data from larger randomized trials.^26,27^ **Figure 6** illustrates some of the possible outcomes that may occur with the addition of multisite pacing or MPP.

Direct LV endocardial pacing may be better than epicardial pacing for CRT.^26^ Endocardial stimulation appears to enhance resynchronization by more rapid activation of the LV. Another potential benefit of endocardial lead placement is the ability to target the site of latest activation. Implementation of endocardial stimulation will require developing safe, effective, and durable instrumentation; introducing reliable and reproducible intraprocedural methods to identify the optimal site of stimulation; and completing controlled trials confirming the superiority of this technique in comparison with standard CRT.^1,27–29^ Three-dimensional mapping and computer modeling will likely play pivotal roles.^1,26^

## Conclusions

CRT is an established option for the management of select patients with systolic HF. Unfortunately, not all CRT recipients respond to this therapeutic modality. In this case series, we have presented five patients who each required additional intervention to achieve CRT responsiveness. Clinicians are encouraged to employ the options presented in **Table 1** and to consider novel, creative approaches in individual nonresponders. We await future developments, such as advances in computer modeling, to further enhance the efficacy and applicability of this valuable device-based intervention.

## Figures and Tables

**Table 1:** Potential Interventions for CRT Nonresponders

• Adding or uptitrating medical therapy
• Changing advice on diet and fluid intake
• Repositioning the LV lead
• Altering atrioventricular or V–V timing (including via novel device-based algorithms)
• His-bundle pacing
• Multisite or multipoint pacing
• Treating arrhythmias that interfere with high-percentage BiV pacing (either medically or invasively)

LV: left ventricular; VV: ventricular–ventricular; BiV: biventricular.
